# Balloon-Assisted Thrombin Injection of a Profunda Femoral Pseudoaneurysm Using Radial Access

**DOI:** 10.14797/mdcvj.1312

**Published:** 2024-01-25

**Authors:** Medhat Chowdhury, Cameron Whilter, Phanindra Antharam, Pritham Reddy, Herman Kado, Matthew Osher

**Affiliations:** 1 Ascension Providence Hospital, Southfield, Michigan, US

**Keywords:** profunda, pseudoaneurysm, balloon assisted, thrombin injection, radial access

## Abstract

The profunda femoral artery is an uncommon location for a pseudoaneurysm and is technically challenging to resolve with traditional techniques, such as ultrasound-guided compression or thrombin injection, owing to its deep anatomical location. Balloon-assisted thrombin injection (BATI) is a technique that has been shown to be effective using contralateral access for technically difficult pseudoaneurysms in high-risk surgical patients. We report a case of BATI using radial access in a patient with a profunda femoral artery pseudoaneurysm.

## Introduction

Pseudoaneurysms (PSAs) are contained ruptures following injury and resultant disruption of the arterial walls that may occur iatrogenically following vascular access, mechanical trauma, or with vascular infections such as mycotic aneurysms.^1^ Post-catheterization PSAs are among the most common complications of diagnostic angiographic procedures and occur in 2% to 6% of interventions.^2,3^ Ultrasound-guided compression (UGC), ultrasound-guided thrombin injection (UGTI), and open surgical repair are potential approaches to management, with UGTI as the preferred approach for post-catheterization PSA.^1^ A recent case report described the successful use of balloon-assisted thrombin injection (BATI) using a radial approach for a technically difficult common femoral artery pseudoaneurysm.^4^ We describe a similar technique for intervention of a profunda femoral artery PSA in a complex surgical patient.

## Case Presentation

A 58-year-old male with past medical history significant for hypertension, morbid obesity (body mass index 41 kg/m^2^), and obstructive sleep apnea underwent an elective endovascular aortic aneurysm repair procedure for a left distal common iliac artery aneurysm measuring 4.1 × 3.9 cm and bilateral internal iliac aneurysms measuring 2.9 cm on the right and 2.5 cm on the left. Despite ultrasound guidance, bilateral femoral artery access was challenging due to the presence of a large pannus and considerable depth of the femoral artery. Balloon-expandable stent grafts were deployed in the aorta extending to bilateral internal iliac and external iliac artery. A completion arteriogram after the procedure revealed no evidence of endoleak and excellent positioning of all devices. Hemostasis at the femoral access sites was attempted using two Perclose sutures (Abbott Cardiovascular). The patient had significant left groin bleeding at the end of the procedure that required femoral cut-down and exploration; no bleeding was noted at the arterial access site, and it was subsequently closed with vicryl sutures.

**Figure 1:**
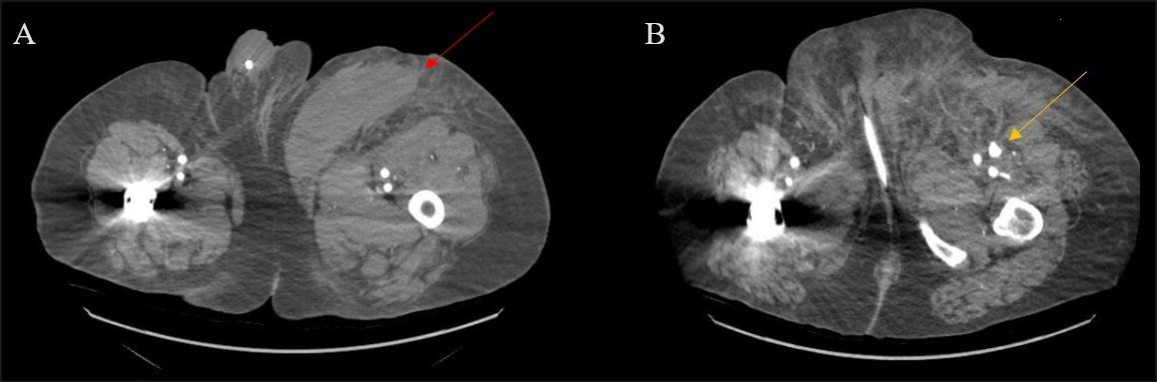
**(A)** 15 × 6 × 5.6-cm left anterior medial thigh hematoma (red arrow). **(B)** 3.7 × 2.1 × 1.6 cm left profunda femoral artery pseudoaneurysm with contrast extravasation (yellow arrow).

On postoperative day 1, the patient was noted to be hypotensive and tachycardic, with a blood pressure of 90/51 mm Hg and a heart rate of 120 beats/min. His physical exam revealed mild tenderness to palpation in the suprapubic region and soft, nontender left and right groin hematomas. Bilateral lower extremities had palpable 2+ posterior tibial and dorsalis pedis pulses. His labs revealed decreases in hemoglobin from 14.9 gm/dL to 12.6 gm/dL and platelets from 290,000/mcL to 119,0000/mcL. Computed tomography angiography (CTA) revealed a 15 × 6 × 5.6-cm left anterior medial thigh hematoma (Figure 1 A) with a 3.7 × 2.1 × 1.6-cm left profunda PSA (Figure 1 B). Given the patient’s body habitus and a large hematoma obscuring sonographic PSA visualization, standard UGTI and UGC were unlikely to be successful. Repeat open exploration and repair was felt to be high risk, therefore surveillance with repeat imaging was pursued. A repeat CTA on postoperative day 3 showed persistence of the bilobed profunda PSA. A multidisciplinary conference involving vascular surgery, interventional radiology, and interventional cardiology was conducted, and the decision was made to proceed with fluoroscopy guided thrombin injection following balloon occlusion of the left profunda femoral artery to prevent distal embolization. Radial-to-peripheral access was planned due to the presence of a new stent graft limiting contralateral up and over access.

## Description of Technique

The left radial artery was accessed with a 6F GLIDESHEATH SLENDER® sheath (Terumo Interventional Systems). A 0.035-cm guidewire was used to introduce a pigtail catheter to the abdominal aorta, and angiography was performed. A 260-cm Terumo Glide Advantage wire was advanced to the left common iliac artery. The left radial sheath was then exchanged for a 149-cm Terumo radial-to-peripheral sheath. Left common iliac angiography demonstrated patent vessels and visualized the PSA arising from the proximal profunda femoral artery.

**Figure 2:**
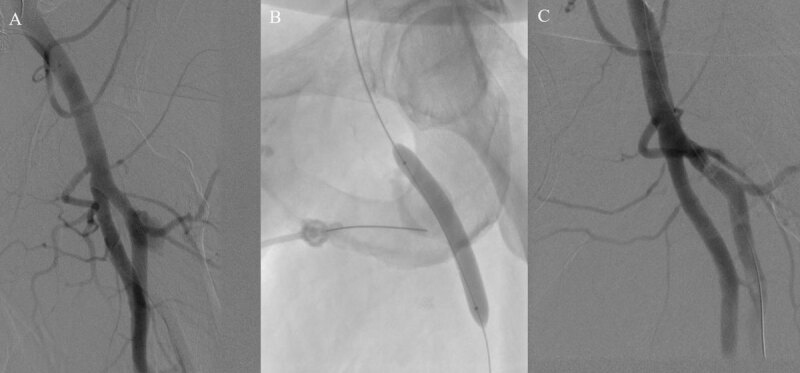
**(A)** Initial angiogram depicting the left profunda femoral artery pseudoaneurysm. **(B)** Imaging showing the position of the needle in the pseudoaneurysm chamber and balloon inflated in the profunda femoral artery to protect against distal embolization. **(C)** Final angiogram showing patency of the superficial and profunda femoral artery and resolution of the pseudoaneurysm.

An over-the-wire 8-mm × 6-cm Metacross balloon (Terumo Interventional Systems) was advanced into the proximal profunda femoral artery and inflated. Contrast injection through the sheath during balloon occlusion demonstrated satisfactory occlusion of the profunda femoral artery with no opacification of the distal segment. Under fluoroscopic guidance, the PSA was accessed with a 22-gauge Chiba needle (Chiba Biopsy Needle 22 g/10 cm) (Figure 2 B). Appropriate positioning was confirmed with aspiration of blood. Contrast injection through the needle demonstrated opacification of the PSA without native vessel extension. The balloon was inflated in the profunda femoral artery and the PSA was embolized with 3,000 international units of thrombin through the Chiba needle.

Postembolization angiography through the sheath confirmed distal patency of the superficial femoral and profunda femoral artery and resolution of the PSA (Figure 2 C). The radial sheath was removed, and hemostasis was achieved with a Terumo radial band. The patient had no postprocedural complications and was successfully discharged on postoperative day 5.

## Discussion

We report a case of successful balloon-assisted thrombin injection using fluoroscopy guidance for a complex patient immediately post vascular surgery. Our case demonstrates a unique approach to the management of profunda PSA using balloon protection via radial artery access.

Profunda PSAs are uncommon owing to the deep anatomic location, and they account for 2% of peripheral artery injuries.^5^ UGTI is the current preferred treatment of choice for most post-catheterization PSAs and has a success rate of 97.5%.^6^ One major complication of UGTI is arterial embolization that may result in limb ischemia, the risk of which is increased by factors PSA with short necks and repeated thrombin injections.^7^ Hence, adequate visualization of the PSA on ultrasound is imperative and was obscured in our patient due to patient habitus and overlying hematoma. In a retrospective review by Elzawy et al. of patients with profunda PSAs, those treated with UGTI required repeat thrombin injections or ultimately endovascular intervention for definitive management.^8^

BATI performed predominantly via a contralateral up and over approach has shown to be safe and 100% efficacious in prior studies.^9^ A contralateral approach was less favorable in our patient due to the inherently higher risk of bleeding, thrombotic occlusion, and transient limb ischemia associated with endovascular graft compared with native artery access.^10^ Hence, we opted for a radial approach to successfully identify and embolize the PSA with thrombin.

## Conclusion

BATI for profunda PSA using radial access is a feasible minimally invasive alternative for patients in whom ultrasound guidance is technically difficult due to obesity.
